# Draft Genome Sequence of the Grapevine Dieback Fungus *Eutypa lata* UCR-EL1

**DOI:** 10.1128/genomeA.00228-13

**Published:** 2013-05-30

**Authors:** Barbara Blanco-Ulate, Philippe E. Rolshausen, Dario Cantu

**Affiliations:** Department of Viticulture and Enology, University of California—Davis, Davis, California, USAa; Department of Plant Sciences, University of California—Davis, Davis, California, USAb; Department of Botany and Plant Sciences, University of California—Riverside, Riverside, California, USAc

## Abstract

The vascular pathogen *Eutypa lata*, which causes Eutypa dieback in grapevines, is a major threat to grape production worldwide. Here, we present the first draft genome sequence of *E. lata* (UCR-EL1). The computational prediction and annotation of the protein-coding genes of UCR-EL1 provide an initial inventory of its potential virulence factors.

## GENOME ANNOUNCEMENT

Eutypa dieback of grapevines is a wood disease caused by the ascomycete *Eutypa lata* (Pers.: Fr.) Tul. & C. Tul. (also known as *E. armeniacae* Hansf. and M. V. Carter) (1, 2). *E. lata* infections result in significant economical losses due to reduced yields, increased crop management costs, and shortened life span of the vines (3, 4).

*E. lata* enters the host through pruning wounds, colonizes the vascular tissues (1, 5), and gradually kills the plant by secreting phytotoxins (6, 7) and cell wall-degrading enzymes (8). Grape cultivars show differences in their susceptibilities to *E. lata* (9), but no resistant cultivars or completely effective management practices are available.

*E. lata* isolate UCR-EL1 was recovered from the margin of a grapevine (*Vitis vinifera* cv. “Cremson”) wood canker collected in Fresno County (California) in 2011. Fungal colony purification and species identification were performed as described by Rolshausen et al. (10). DNA was extracted using a modified cetyltrimethylammonium bromide (CTAB) method (11), and 7.3 Gb of Illumina HiSeq 2000 sequence data was generated. Most (99.77%) of the 62.3 million quality-trimmed (*Q* ≥ 30) and contaminant-filtered reads were assembled using CLC Genomic Workbench v6.0; 2,334 scaffolds (3,322 contigs; median coverage, 97×) with total length of 54.0 Mb (N_50_, 68.3 kb; L_50_, 238; gaps, 103 kb; G+C content, 46.6%) were assembled. Assembly parameters were optimized to achieve maximal assembly completeness of the gene space estimated using the Core Eukaryotic Genes Mapping Approach (CEGMA) analysis (12). By mapping 248 low-copy core eukaryotic genes (CEGs) (12), which are conserved across higher eukaryotes, the UCR-EL1 genome was estimated to be >97% complete.

Scaffolds were masked for repeats using RepeatMasker (13), and gene prediction was performed with the eukaryotic gene finder Augustus (14), trained using the gene models identified by CEGMA (12). A total of 11,818 complete protein-coding sequences were obtained, which is similar to the gene content of other ascomycetes (15, 16). Ninety-two percent of the predicted proteome was annotated based on its sequence homology to proteins in the NCBI nonredundant (nr) database (BLASTp, e-value ≤10^-3^). While these *ab initio*-discovered gene models need to be further curated and validated using empirical transcript data, they provide us with a first glimpse of the functions encoded in the *E. lata* genome. In agreement with the known capability of *E. lata* to degrade woody tissues (8), we found among the 1,224 potentially secreted proteins (SignalP v4.0 [17]) a rich repertoire of cell wall-degrading enzymes comprising 217 putative glycoside hydrolases annotated based on homology with proteins in the CAZy database (18). The most abundant CAZy families identified among the putative secreted proteome were GH61 (26 genes), GH43 (22 genes), and GH16 (17 genes). While GH61 enzymes enhance the breakdown of lignocellulosic material in combination with cellulolytic enzymes (19), GH43 and GH16 enzymes have hemicellulolytic activities. A large number of putative cytochrome P450 monooxygenases (205 genes), known to be involved in lignin oxidation, were also found, as is reported in other genomes of wood-rotting fungi (20–22).

### Nucleotide sequence accession numbers.

This Whole-Genome Shotgun project has been deposited at DDBJ/EMBL/GenBank under the accession no. AORF00000000. The version described in this paper is the first version, accession no. AORF01000000.
